# Genome Sequence of the Brown Rot Fungal Pathogen Monilinia laxa

**DOI:** 10.1128/genomeA.00214-18

**Published:** 2018-04-26

**Authors:** Miguel A. Naranjo-Ortíz, Silvia Rodríguez-Píres, Rosario Torres, Antonieta De Cal, Josep Usall, Toni Gabaldón

**Affiliations:** a Centre for Genomic Regulation (CRG), The Barcelona Institute of Science and Technology, Barcelona, Spain; b Universitat Pompeu Fabra (UPF), Barcelona, Spain; c Department of Plant Protection, Instituto Nacional de Investigación y Tecnología Agraria y Alimentaria (INIA), Madrid, Spain; d IRTA, XaRTA-Postharvest, Lleida, Catalonia, Spain; e ICREA, Barcelona, Spain

## Abstract

Monilinia laxa (phylum Ascomycota) is a plant pathogen responsible for the brown rot blossom blight disease in stone fruit trees of the Rosaceae family, such as apricots. We report here the genome sequence of strain 8L of this species, which was assembled into 618 scaffolds, having a total size of 40.799 Mb and encoding 9,567 unique protein-coding genes.

## GENOME ANNOUNCEMENT

Brown rot caused by Monilinia laxa (Aderhold and Ruhland) honey, M. fructicola (winter) honey, or M. fructigena (Aderhold and Ruhland) is a serious fungal disease in the main production areas of stone fruits around the world, causing bud and shoot wilt, cankers on branches, and fruit rot (1). The biggest losses occur in the fruit, with levels reaching up to 80% in years with favorable weather for disease development (2, 3). Although fruits with symptoms may appear in the field, the greater amount of rot appears during postharvest, causing great economic impact for the producer.

M. laxa isolate 8L was obtained from the culture collection of the Plant Protection Department of the Instituto Nacional de Investigación y Tecnología Agraria y Alimentaria (INIA), Madrid, Spain. This isolate was originally collected on mummified fruit from a commercial plum orchard (cv. Sungold) in Lagunilla, Salamanca, Spain, in 2015. A single spore of this strain was grown on potato dextrose agar amended with tomato (PDA-T) at 22°C for 7 days. Spore suspensions from 7-day-old PDA-T plates were incubated in an orbital shaker at 150 rpm at 22°C in the dark for 24 h. The mycelium was harvested by filtration through Whatman filter paper and freeze dried. DNA was obtained by phenol-chloroform-isoamyl alcohol extraction and isopropanol precipitation and digested with DNase-free RNase A (65°C, 10 min). Sequencing was performed with Illumina HiSeq2000 sequencing technology using paired-end and mate-pair libraries. Genome assembly was performed using dipSPADES from the SPAdes version 3.9.0 software (4) with default settings. For this step, only the paired-end library was used. Prior to this, all libraries were trimmed using Trimmomatic version 0.36 (5). Scaffolding was done with SSPACE version 3.0 (6) with default settings and employing both libraries. Scaffolds with a size below 1 kb were removed. The final version contained a total scaffold number of 619, an *N*_50_ value of 124.845 kb, and a total genome size of 40.799 Mb. Gene prediction was done using Augustus version 2.7 (7) with Botrytis cinerea (Botryotinia fuckeliana) as the model species, which predicted a total of 9,567 putative protein-coding genes, from which 598 are orphans (6.25%). In order to assess the completeness of our gene prediction, we used BUSCO (8) with a data set consisting of pezizomycotina single-copy ortholog genes (3,156 genes). We retrieved a total of 2,970 completed BUSCO orthologs (94.1%), 63 fragmented BUSCO orthologs (2.0%), and 123 missing BUSCO orthologs (3.9%). We used the PhylomeDB pipeline (9) to compare our genome with that of 12 other species in the class Dothideomycetes. This comparison shows that M. laxa presents a smaller proteome compared with the related crop pathogens Botryotinia fuckeliana and Sclerotinia sclerotiorum. This is consistent with the narrower host range in M. laxa compared to the other two pathogens, a situation similar to other groups of plant necrotrophic pathogens such as those belonging to the genera Penicillium (10, 11) and Colletotrichum (12).

### Accession number(s).

The whole-genome shotgun project reported here has been deposited at GenBank under the accession number PTPU00000000 (BioProject number PRJNA433296) and corresponds with the first version. The genome’s PhylomeDB accession code is 801. The strain Monilinia laxa 8L has been deposited in the Spanish Culture Type Collection with the number CECT 21100 and has synonyms ML8L and LASA2015MOA38.
